# Computational Analysis of *Ca*^2+^ Oscillatory Bio-Signals: Two-Parameter Bifurcation Diagrams

**DOI:** 10.3390/e23070876

**Published:** 2021-07-08

**Authors:** Wieslaw Marszalek, Jan Sadecki, Maciej Walczak

**Affiliations:** Department of Computer Science, Opole University of Technology, 45-758 Opole, Poland; j.sadecki@po.edu.pl (J.S.); maciej.k.walczak@gmail.com (M.W.)

**Keywords:** periodic dynamics verification, cytosolic calcium oscillations, two-parameter bifurcations, period-n solutions, frequency diagrams, chaos

## Abstract

Two types of bifurcation diagrams of cytosolic calcium nonlinear oscillatory systems are presented in rectangular areas determined by two slowly varying parameters. Verification of the periodic dynamics in the two-parameter areas requires solving the underlying model a few hundred thousand or a few million times, depending on the assumed resolution of the desired diagrams (color bifurcation figures). One type of diagram shows *period-n* oscillations, that is, periodic oscillations having *n* maximum values in one period. The second type of diagram shows frequency distributions in the rectangular areas. Each of those types of diagrams gives different information regarding the analyzed autonomous systems and they complement each other. In some parts of the considered rectangular areas, the analyzed systems may exhibit non-periodic steady-state solutions, i.e., constant (equilibrium points), oscillatory chaotic or unstable solutions. The identification process distinguishes the later types from the former one (periodic). Our bifurcation diagrams complement other possible two-parameter diagrams one may create for the same autonomous systems, for example, the diagrams of Lyapunov exponents, $L^{s}$ diagrams for mixed-mode oscillations or the 0–1 test for chaos and sample entropy diagrams. Computing our two-parameter bifurcation diagrams in practice and determining the areas of periodicity is based on using an appropriate numerical solver of the underlying mathematical model (system of differential equations) with an adaptive (or constant) step-size of integration, using parallel computations. The case presented in this paper is illustrated by the diagrams for an autonomous dynamical model for cytosolic calcium oscillations, an interesting nonlinear model with three dynamical variables, sixteen parameters and various nonlinear terms of polynomial and rational types. The identified frequency of oscillations may increase or decrease a few hundred times within the assumed range of parameters, which is a rather unusual property. Such a dynamical model of cytosolic calcium oscillations, with mitochondria included, is an important model in which control of the basic functions of cells is achieved through the $Ca^{2+}$ signal regulation.

## 1. Introduction

### 1.1. Preliminaries

Periodic cycles—solutions of nonlinear planar (two variables) systems—are covered by the celebrated Poincaré–Bendixson theorem proven (under certain assumptions) with the use of the Green theorem [1]. To verify an absence of limit cycles, one may use the Dulac’s criterion [2]. Such an approach becomes problematic for multi-variable nonlinear systems having a wide spectrum of possible steady-state responses that change (bifurcate) with slowly varying parameters. Verification of periodic (or oscillatory chaotic) responses in such cases is done by numerical methods, often with a parallel computing approach [3,4]. Determining bifurcations of nonlinear dynamical systems often require a sophisticated computational approach to visualize the obtained results [5,6].

In this paper, we deal with the computer-aided verification of oscillatory responses and qualitative analysis of nonlinear dynamical systems [7]. An application case study is presented for a rather intriguing cytosolic (with mitochondria) calcium oscillatory dynamical model having numerous nonlinear terms and interesting properties, one of which is a wide change of frequency of oscillations when parameters vary. We compute two-parameter bifurcation diagrams of that system to identify areas of periodic and non-periodic steady-state responses.

### 1.2. The Reliability Issue of the Numerically Computed Periodic and Chaotic Solutions

One of the major reasons of writing this paper follows from the well-documented issue of the reliability of the chaotic and periodic solutions obtained with finite precision computations. The central issue is the fact that obtaining entirely identical long-term chaotic solutions via numerical solvers is practically impossible even when the same algorithm, time step and initial conditions are applied to a particular nonlinear ordinary differential equation (ODE) system. Reports on such an issue with interesting findings for the well-known chaotic systems are discussed in [8]. The concept of critical time, $T_{c}$, has been introduced, to mark the time instant beyond which two chaotic solutions depart from each other to become completely different. This has been further expanded in [9,10] and the best description of the problem when dealing with chaotic solutions is the following quotation from [9]: “…even when the initial conditions are exactly the same, the algorithm is not changed and the step time is kept unchanged, one can find multiple pseudo-orbits simply by changing the way the model is written” and further in the same paper: “The question that arises (…) is: with the same initial condition, same scheme of discretization and equivalent set of differential equations, which orbit is the true one? This is not an easy question to answer since there is almost no option but to use finite precision machine.” In [8], the following observation is made: “Parker and Chua [11] pointed out that a ‘practical’ way of judging the accuracy of numerical results of a non-linear dynamic system is to use two (or more) ‘different’ routines to integrate the ‘same’ system: the initial time interval over which the two results agree is then ‘assumed’ to be accurate and predictable. More precisely speaking, the computed results beyond the critical decoupling time $T_{c}$ are not reliable.”

The above issues are, to some extent, related to the results presented in the present paper, in which we follow the guidance expressed in the above quotations, and study two different bifurcation 2D diagrams (of n-period and frequency types) for one particular non-linear model with complex $Ca^{2+}$ oscillatory solutions. Our goal was to identify and predict periodic and chaotic solutions using those two types of diagrams. Within the n-period type of 2D diagrams, we differentiate our calculations by using three maximum values of n (64, 128 and 182; see Section 3). To further modify our approach and numerical algorithms, we used two other tools to identify periodic and chaotic oscillations, namely the sample entropy and 0–1 test for chaos (Section 5). Throughout the paper, we compare the obtained results (2D diagrams) to conclude that the same types of solutions are obtained no matter which tool (type of diagram) is used. However, we would also like to point out that the issues raised in [8,9,10,11] are not fully relevant to our study. Even when one obtains two different chaotic solutions and attractors beyond the critical time $T_{c}$, such solutions will still be identified as chaotic. Thus, our diagrams will stay the same, and the color indicating particular response will not change (see Section 2 and Section 3). The 0–1 test for chaos (see Section 5) will still result in a number close to 1 indicating chaotic responses.

### 1.3. Autonomous System of Calcium Oscillations and Its Basic Properties

The slowly varying parameters of nonlinear dynamical systems described by ODEs may cause significant changes to the systems’ steady-state responses. Typical visualization of those changes when one parameter varies slowly has the form of one-parameter bifurcation diagrams (figures) with the varying parameter representing the horizontal axis while the vertical axis is a certain quantity characterizing the changing responses. That quantity could be the identified maximum values of the response in a chosen time interval, the output of the 0–1 test for chaos (a number between 0 (for periodic responses) and 1 (for chaotic ones)), the frequency of periodic output, entropy or others [4]. In this paper, we illustrate our bifurcation diagrams (mostly with two slowly varying parameters) with the use of an interesting autonomous system described in Appendix A. The system (A3) has been used in the literature for a qualitative analysis of the oscillatory cytosolic calcium responses [12,13,14,15,16,17,18].

As a result of the many nonlinear terms, a computational approach to analyze is (A3), but some analytical analysis is also possible [12]. Taking into account the structure of the right-hand functions ${\tilde{f}}_{i}$, i=1,2,3, in (A3), it can be shown that for the non-negative initial conditions, the three solutions, $x_{i}\left(t\right)$ are all non-negative and bounded. Furthermore, by denoting the equilibrium points of the three variables by $\left({\bar{x}}_{0},{\bar{y}}_{0},{\bar{z}}_{0}\right)$, one can compute $x_{3}$ from ${\tilde{f}}_{3}$, substitute it into ${\tilde{f}}_{2}$ to express $x_{2}$ as a function of $x_{1}$ and then ${\tilde{f}}_{1}$ can be transformed into a polynomial equation in ${\bar{x}}_{0}$ only (with the parameters *p*). Fixing 15 out of possible 16 parameters and considering the remaining parameter as slowly varying, we can establish a dependence of ${\bar{x}}_{0}$ on that one varying parameter. Moreover, the linearization of (A3) along ${\bar{x}}_{0}$ will yield a Jacobian with the characteristic polynomial of the form [13]

(1) $$P\left(\lambda ,\bar{p}\right)=\lambda^{3}+a_{1}\left(\bar{p}\right)\lambda^{2}+a_{2}\left(\bar{p}\right)\lambda +a_{3}\left(\bar{p}\right),$$

which can further be used to analyze the stability properties, and, for example, to find the Hopf bifurcation points. Such an analysis is not very relevant to the topic of this paper, therefore it is omitted here.

**Figure 1:**
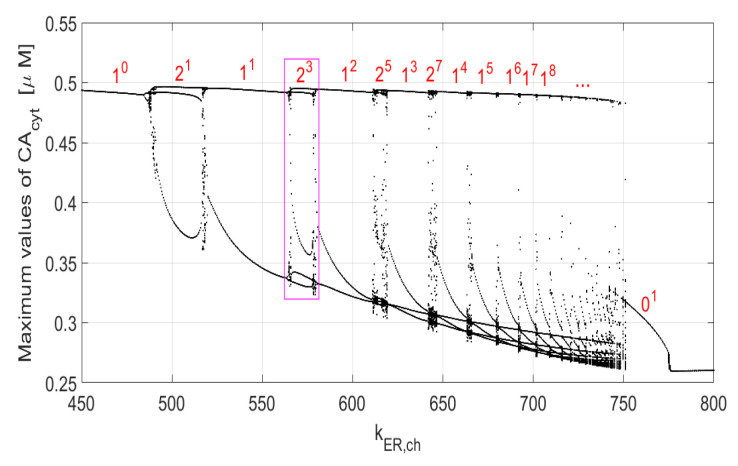
Bifurcation diagram of (A3) for $K_{ch}=2.1$.

**Figure 2:**
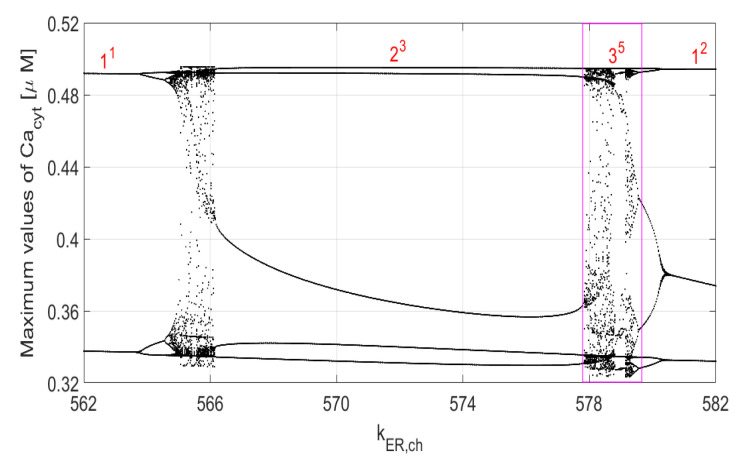
Bifurcation diagram of (A3) for $K_{ch}=2.1$ (rectangle $2^{3}$ in Figure 1). Note that $3^{5}=2^{3}⨁1^{2}$.

Figure 1 illustrates one of the many one-parameter bifurcation diagrams of (A3) when $k_{ER,ch}$ is the slowly varying parameter and the vertical axis shows the values of maximum points identified in one period of the steady-state solution of the system. The $L^{s}$ notation (with *L* and *s* being co-prime integers) indicates a mixed-mode periodic oscillation. *L* and *s* are the numbers of *large* and *small* maximum values of oscillations in one period. For example, the $2^{3}$ mixed mode oscillation in an interval around $k_{ER,ch}=570$ has 2 *large* maximum values (close to each other) around the value of 0.49 and three *small* maximum values around 0.33–0.36. The diagram in the marked rectangle in Figure 1 is shown in more detail in Figure 2. Note an interesting property of (A3)—the $L^{s}$ periodic responses form a Farey sequence of co-prime integers. For example, $2^{3}$ is formed from the two neighboring sequences $1^{1}$ and $1^{2}$, with $2^{3}=1^{1}⨁1^{2}$, where ⨁ is the Farey addition. In general, $L^{s}=L_{1}^{s_{1}}⨁L_{2}^{s_{2}}$, with $L=L_{1}+L_{2}$ and $s=s_{1}+s_{2}$. See [14] for more details and the relation of the $L^{s}$ type of oscillations to the Ford circles, Stern–Brockot tree, sequences of *firing* numbers and the Riemann zeta function. The $L^{s}$ mixed-mode periodic oscillations have also been considered in the context of two-parameter bifurcation diagrams for the modified Chua’s circuits in [6]. The intermingling intervals of periodic and chaotic solutions (as in Figure 1 and Figure 2) can also be used to determine fractal dimension of (A3) [14]. We chose to vary the same parameters of (A3) that were used in [12,15,16,17,18] for their one-parameter bifurcation analysis. The remaining parameters are constant in our 2D bifurcation analysis, as described in Appendix A.

### 1.4. Bifurcation Diagrams of Period-n Responses and Frequency Distribution

In the next section, we use the $L^{s}$ type of periodic oscillations as *(L+s)-periodic* oscillations, indicating that in one period there are L+s maximum values. Thus, the two-parameter diagrams of *n* maximum values, n=1,2,…,64, presented next, indicate that the solutions are periodic, with n=L+s. Thus, no distinction is made to differentiate between *large* and *small* maximum values. For this reason, two different $L_{1}^{s_{1}}$ and $L_{2}^{s_{2}}$ oscillations are both of the same *period-n* type, if $L_{1}+s_{1}=L_{2}+s_{2}$. From the point of view of *period-n* oscillations, we have a possibility of n+1 different types of oscillations, called the *period-n* ones, as, for example, the following six *period-5* oscillations may come from the $5^{0}$, $4^{1}$, $3^{2}$, $2^{3}$, $1^{4}$ and $0^{5}$ mixed-mode oscillations. All these six mixed-mode oscillations are marked by the same color and the number n=5 in the two-parameter bifurcation diagrams of the number of maximum values in one period.

The above *period-n* oscillations as illustrated in Figure 1 and Figure 2 (or in the two-parameter diagrams in the next section) do not provide any information as far as the frequency of oscillations is concerned. Once a *period-n* oscillation is established, an extra computational work can be performed to measure the frequency and, thus, create one- or two-parameter frequency diagrams. Both, the *period-n* and frequency diagrams complement each other to provide a better understanding of how nonlinear dynamical systems behave when their parameters vary. The frequency diagram may be essential in other kinds of analysis. For examples, it has been established recently that applying the 0–1 test for chaos may require a prior knowledge of the maximum frequency of the continuous spectrum of chaotic signals to properly choose certain parameters used in that test [19,20].

Depending on the ranges of varying parameters, it often happens that the same type of *period-n* oscillations differ significantly in frequencies, as we have discovered by creating diagrams in the next section. The difference could be by a factor of a few hundred times, as it happens for the system (A3). Further discussion on the range of frequency changes in the calcium oscillatory systems can be found in [21].

The wide range of frequencies of oscillations of (A3) cannot be seen from the diagrams in Figure 1 and Figure 2 or other similar one-parameter diagrams, but it is known that (A3) has that peculiar feature of a wide range of frequency change and rather small amplitude change, even with a small interval of the varying parameters, including $k_{ER,ch}$ (“In some cells, time intervals between two $Ca^{2+}$ spikes change from a few seconds to several minutes or even tens of minutes. However, in contrast to the large changes in frequency, the amplitude of $Ca^{2+}$ oscillations remains nearly constant in many cell types. This likely allows the cell to encode information in the frequency of $Ca^{2+}$ oscillations.” See [16]).

## 2. Two-Parameter Period-n Bifurcation Diagrams

**Figure 3:**
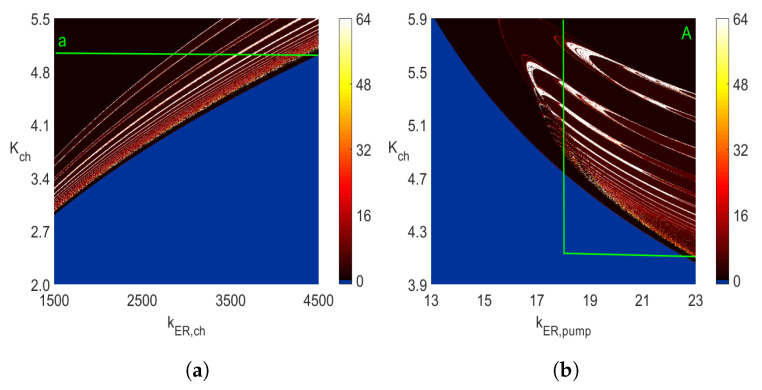

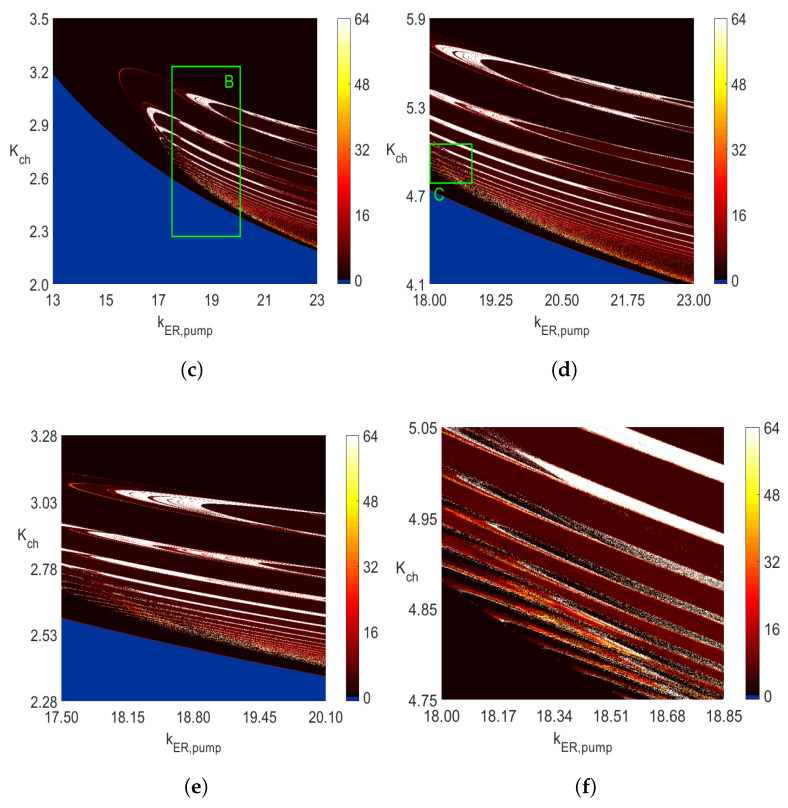
Two-parameter 600×600 diagrams of the number of maximum values identified in one period, each obtained by using the Runge–Kutta IV solver with dt=0.0005 for 0≤t≤1000 when the solutions in the interval 500≤t≤1000 were used in the identification. Obtaining each of the above two-parameter diagrams requires solving (A3) $3.6\times 10^{5}$ times. (**a**) Diagram with $k_{ER,pump}=20$. (**b**) Diagram with $k_{ER,ch}=3500$. (**c**) Diagram with $k_{ER,ch}=1000$. (**d**) Diagram in area *A* in subfigure b. (**e**) Diagram in area *B* in subfigure c. (**f**) Diagram in area *C* in subfigure d.

Figure 3 presents two-parameter bifurcation diagrams showing *period-1* to *period-63* oscillations. The blue color indicates areas with constant steady-state solutions, that is, starting with the zero-initial conditions, all solutions of (A3) end up at the equilibrium points. Additionally, there is a degree of ambiguity with the white color associated with the number 64 in the color vertical bars. Namely, all periodic solutions with 64 (or more) maximum values in one period and all chaotic responses are marked with the number 64 (white color). The number 64 has no particular significance, and the computations can be done with a higher upper bound than 64. Notice that the diagram in rectangle *A* in Figure 3b is enlarged in Figure 3d. Rectangle *B* in Figure 3c is enlarged in Figure 3e, while the diagram in rectangle *C* in Figure 3d is enlarged in Figure 3f. To create each of the diagrams in Figure 3, we solved (A3) 360,000 times as 600 × 600 discrete points were used. As in the case of one-parameter diagrams in Figure 1 and Figure 2, one cannot deduct any information about the frequency of *period-n* oscillations from the diagrams in Figure 3. As explained before, each discrete point with n=1,…,63 maximum values in one period indicates the sum L+s(=n) rather than the *L* and *s* separately. Of course, the maximum values of the analyzed variable in one period cannot be retrieved from the diagrams in Figure 3. On the other hand, the periodic oscillations with n≥64 and chaotic areas represented by the white color in Figure 3 are identifiable in the same way one can identify them in one-parameters diagrams. Those are rather narrow intervals with multiple dots in vertical lines distributed between the lower and upper maximum values seen in Figure 1 and Figure 2, for example, for the $k_{ER,ch}$ values less than 566 and around 578 in Figure 2.

## 3. Two-Parameter Frequency Distribution Diagrams

**Figure 4:**
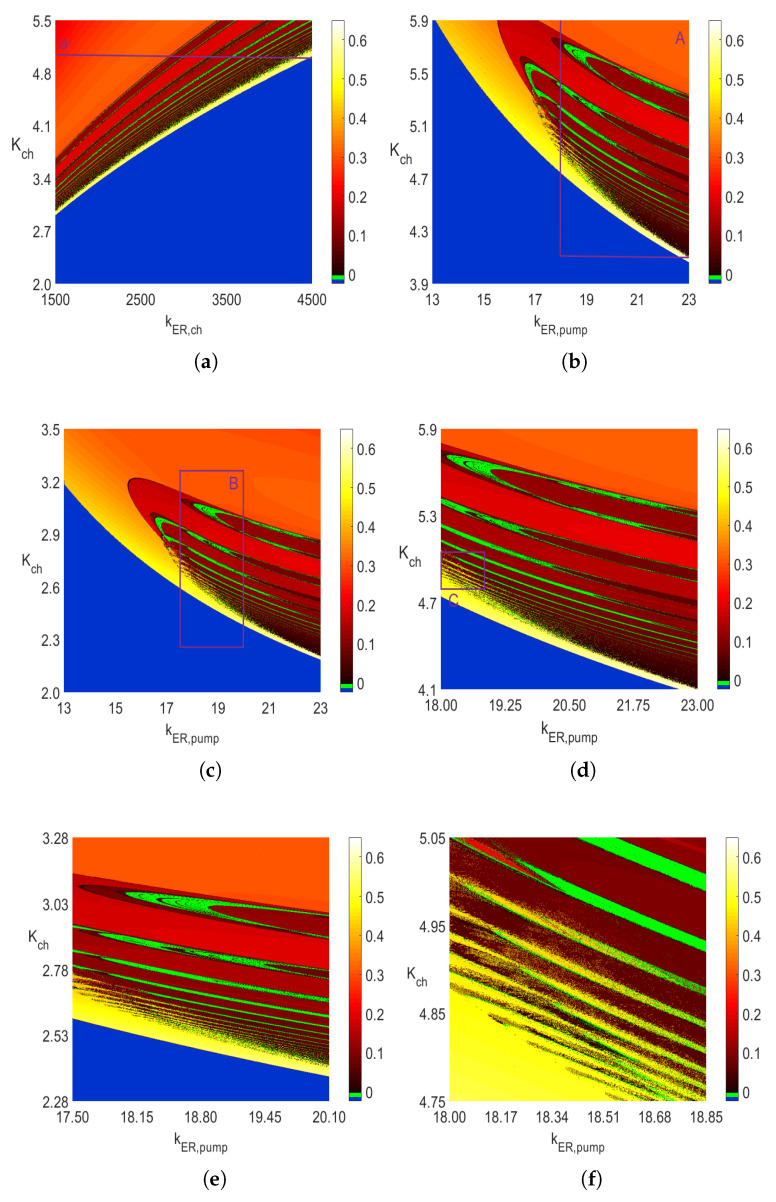
Two-parameter 600×600 diagrams of the frequency of periodic oscillations, each obtained by using the Runge-Kutta IV solver with dt=0.0005 for 0≤t≤1000 when the solutions in the interval 500≤t≤1000 were used in the identification. Obtaining each of the above two-parameter diagrams requires solving (A3) $3.6\times 10^{5}$ times. No frequency values are assigned to the blue and green areas. (**a**) Diagram with $k_{ER,pump}=20$. (**b**) Diagram with $k_{ER,ch}=3500$. (**c**) Diagram with $k_{ER,ch}=1000$. (**d**) Diagram in area *A* in subfigure b. (**e**) Diagram in area *B* in subfigure c. (**f**) Diagram in area *C* in subfigure d.

Figure 4 presents frequency distribution of the periodic solutions of (A3) in the same rectangular areas as the *period-n* bifurcation diagrams in Figure 3. Again, the blue color has the same meaning as that explained before for the blue areas in Figure 3. The extra green color in Figure 4 is not associated with any frequency value as it represents the solutions of either *period-n* oscillations for n≥64 or chaotic solutions for which, obviously, one cannot find frequency. This means that the green areas in Figure 4 correspond to the white areas in Figure 3.

**Figure 5:**
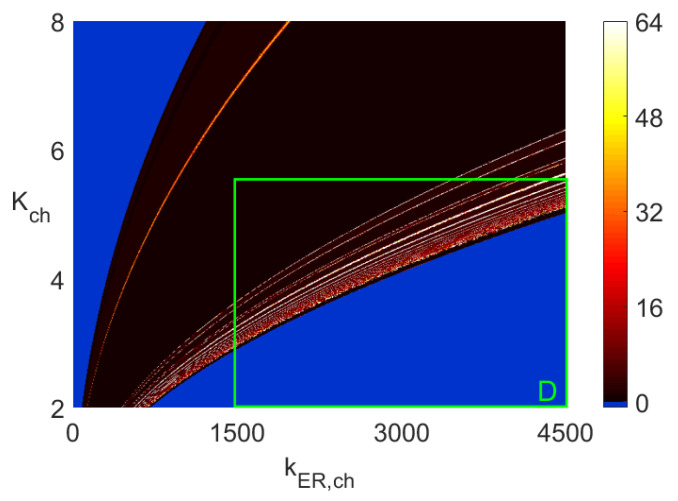
Maximum values diagram of (A3).

**Figure 6:**
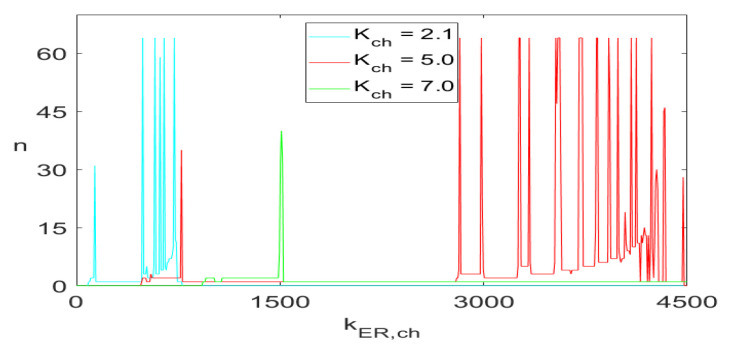
The *n* values of (A3) as functions of $k_{ER,ch}$ for $K_{ch}=\left\{2.1,5.0,7,0\right\}$.

**Figure 7:**
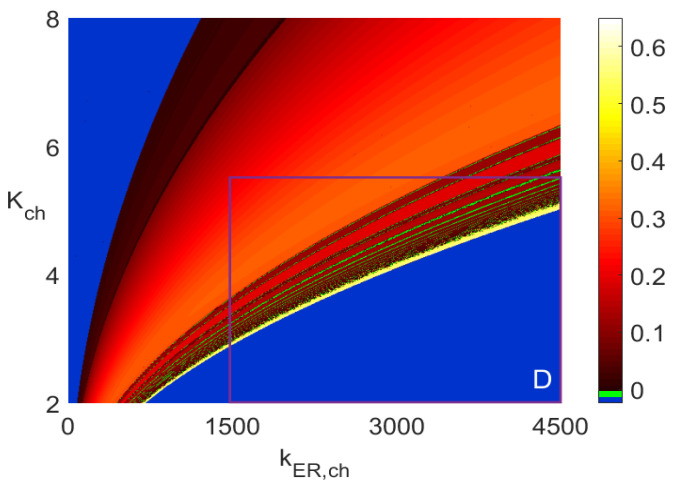
Frequency bifurcation diagram of (A3).

**Figure 8:**
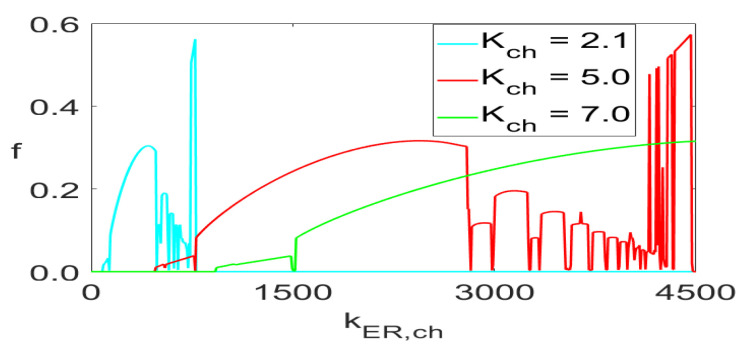
Frequency values of (A3) as functions of $k_{ER,ch}$ for $K_{ch}=\left\{2.1,5.0,7,0\right\}$.

Furthermore, the diagrams in the larger two-parameter rectangles in Figure 5 (with an accompanying Figure 6) and Figure 7 (with an accompanying Figure 8) are the *period-n* and frequency distribution diagrams, respectively. The smaller rectangles *D* in those figures are the rectangles in Figure 3a and Figure 4a, respectively. The purpose of creating Figure 5 and Figure 7 is two-fold. First, notice that the central part of the diagram in Figure 5 is dark brown, indicating mostly *period-1* and *period-2* oscillations.

However, from Figure 7, it follows that some of those oscillations have frequencies that differ significantly. Notice the very dark brown area next to the blue area on the left side in Figure 7. The frequencies there are as low as 0.003 Hz. Similar *period-1* frequencies in the very narrow yellow strip next to the blue area in the lower right corner are around 0.6 Hz. Thus, the frequencies of the same type of *period-1* oscillations differ by the factor of around 200. Selecting a point on the line *a* for $k_{ER,ch}=4400$ in Figure 3a indicates *period-1* oscillations to which we assign the corresponding point on line *a* in Figure 4a on the narrow yellow band with frequency around 0.6 Hz. Similar significant change in the frequency values can be observed between the yellow and brown areas in the lower right corner areas in Figure 4b–d. This confirms the second reason for the usefulness of the two-parameter diagrams: they give a broader view of the possible changes as far as the types of *period-n* solutions and their corresponding frequencies are concerned.

Figure 6 and Figure 8 show one-parameter diagrams of the *n* values and frequency, respectively, as functions of $k_{ER,ch}$ for three values of $K_{ch}$. Those are simply horizontal cross-cuts of Figure 5 and Figure 7. The narrow intervals with spikes at n=64 in Figure 6 indicate intervals of $k_{ER,ch}$ yielding white bands in Figure 5 and green bands in Figure 7. Similarly, the frequency spikes in Figure 8, reaching the values close to 0.6 Hz, correspond to the points in a narrow yellowish-white band close to the blue area in the lower right corner in Figure 7.

The two types of the two-parameter diagrams for (A3) are not the only ones that can be created for the same autonomous system. It is certainly possible to compute the two-parameter diagrams for Lyapunov exponents [22], stability domains [23], Poincaré return maps [24], entropy [25] and the 0–1 test for chaos [26]. The later two-parameter diagrams for the electric circuits, Lorenz and Rössler chaotic systems, are presented in [20,27,28].

**Figure 9:**
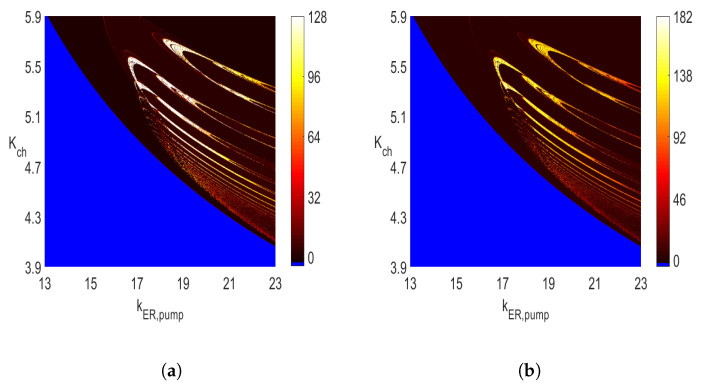
Two-parameter 600 × 600 diagrams of the number of maximum values identified in one period with (**a**) max(n)=128 and (**b**) max(n)=182. See Figure 3b for comparison and other parameters. (**a**) Diagram as in Figure 3b. (**b**) Diagram as in Figure 3b.

In addition, notice that while the maximum value of *n* used in the diagrams in Figure 3 is 64, the character of the diagrams is preserved when that value is increased. For example, Figure 9a,b show how the diagram from Figure 3b changes when the maximum value of *n* is increased to 128 (Figure 9a) and 182 (Figure 9b). The reduction of the amount of pure white color with the increased maximum of *n* is clearly noticeable. This indicates that for many of those pairs of parameters of $\left(K_{ch},K_{ER,pump}\right)$, for which n=128 (the white color in Figure 9a), the color changes to yellow in Figure 9b. There are rather few pairs of parameters $\left(K_{ch},K_{ER,pump}\right)$ with *n* values close to 182, as shown in Figure 9b. The sharp jump of *n* from single digits (i.e., n<10 represented by the brown color) to high values (i.e., *n* around 182 represented by the white color) is an indicator of the changes in oscillatory behavior similar to a periodic to chaotic bifurcation or periodic to bursting transition. This happens, for example, around the area of $\left(K_{ch},k_{ER,pump}\right)=\left(5.5,17\right)$ in Figure 3b and Figure 9a,b.

## 4. Details of Computations of the Two-Parameters Bifurcation Diagrams

**Figure 10:**
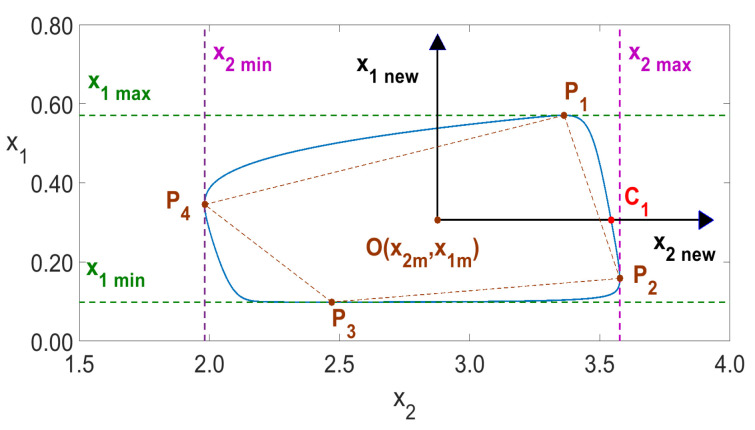
Period-1 solution of (A3) for $K_{ch}=10$.

**Figure 11:**
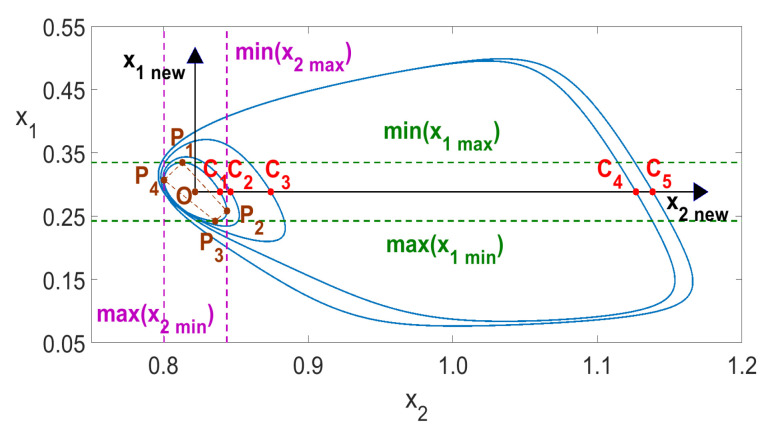
Period-5 solution of (A3) for $K_{ch}=5.15$.

Generally, if an oscillation system has periodic solution then for any two variables, say $x_{i}\left(t\right)$ and $x_{j}\left(t\right)$, we have a homoclinic orbit $F(x_{i},x_{j})=0$ in the form of closed loops. The number of loops *n* is equal to the number of local maxima (or minima) of both $x_{i}\left(t\right)$ and $x_{j}\left(t\right)$ occurring within one period, as shown in Figure 10 for n=1 and in Figure 11 for n=5.

To identify the number *n*, it is first necessary to determine the geometrical center of the innermost loop, denoted by $O(x_{2m},x_{1m})$ in Figure 10 and Figure 11 for the variables $x_{1}$ and $x_{2}$. It is well-known that for any two-dimensional area *A* in variables *x* and *y*, the center is determined by the double integrals

(2) $$x_{m}=\frac{\int \int x(dA)}{\int \int dA},\;y_{m}=\frac{\int \int y(dA)}{\int \int dA}.$$

When we consider the most inner loop (the loop of the smallest area), then an approximate method to find the center of that loop uses the four points $P_{1},\ldots ,P_{4}$ defining the quadrangle $P_{1}P_{2}P_{3}P_{4}$, as is shown in Figure 10 and Figure 11. The center of that quadrangle has been determined by the values $max(x_{2min})$, $min(x_{2max})$, $max(x_{1min})$ and $min(x_{1max})$ (see Figure 11), where, in general, $max(x_{jmin})$ denotes the largest local minimum of $x_{j}\left(t\right)$, while $min(x_{jmax})$ denotes the smallest local maximum of $x_{j}\left(t\right)$. As the computations showed, the above method of determining the center *O* proved to be accurate. To avoid any transients, such an analysis was carried out in this paper in the interval of identification [500,1000] seconds, while the solution of (A3) was computed in the interval 0≤t≤1000.

**Figure 12:**
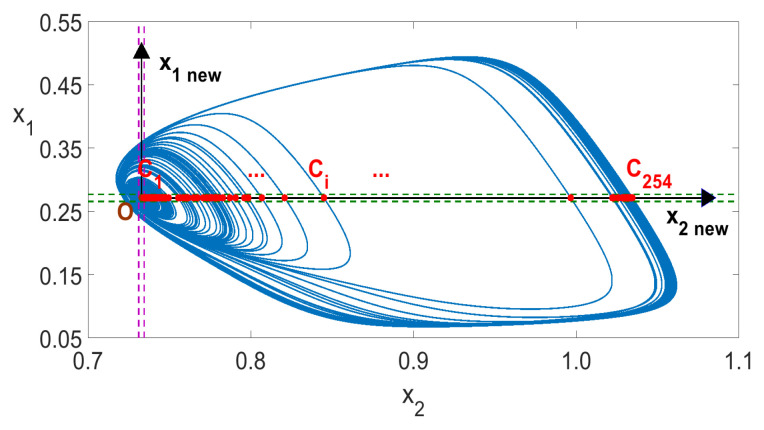
Chaotic solution of (A3) for $K_{ch}=4.683$.

The number of intersections of the graph $F(x_{i},x_{j})=0$ with the positive half axis $x_{2new}$ (the points $C_{i}$ in Figure 10 and Figure 11) is exactly equal to the number of local maxima of $x_{2}\left(t\right)$ in one period. The solution values of x(t) are computed at discrete points. The intersections values (coordinates of the points $C_{i}$) were obtained as a result of linear interpolation, based on the two nearest discrete values (below and above the $x_{2new}$ axis). The value of the time variable *t*, for which the intersections with the positive half axis $x_{2new}$ happen, is determined analogously by linear interpolation. The period $T_{0}$ was determined as $T_{0}=t_{i+n}-t_{i}$, where *n* is the number of different intersections of the positive half axis of $x_{2new}$. We assumed in this paper that the intersections of the positive half axis $x_{2new}$ are considered identical if the values obtained by linear interpolation do not differ by more than the assumed certain error value ϵ. We assumed ϵ=0.0001 for all computations of both one- and two-parameter diagrams in this paper. When a chaotic response is obtained, then the calculations of both *n* and $T_{0}$ are not possible, as no homoclinic orbits occur, as illustrated in Figure 12.

The number of intersections of the graph $F(x_{i},x_{j})=0$ with the positive half axis $x_{2new}$ (the points $C_{i}$ in Figure 10 and Figure 11) is exactly equal to the number of local maxima of $x_{2}\left(t\right)$ in one period. The solution values of x(t) are computed at discrete points. The intersection values (coordinates of the points $C_{i}$) were obtained as a result of linear interpolation, based on the two nearest discrete values (below and above the $x_{2new}$ axis). The value of the time variable *t* for which the intersections with the positive half axis $x_{2new}$ happen, is determined analogously by linear interpolation. The period $T_{0}$ was determined as $T_{0}=t_{i+n}-t_{i}$, where *n* is the number of different intersections of the positive half axis of $x_{2new}$. We assumed in this paper that the intersections of the positive half axis $x_{2new}$ are considered identical if the values obtained by linear interpolation do not differ by more than the assumed certain error value ϵ. We assumed ϵ=0.0001 in all computations of both one- and two-parameter diagrams in this paper. When a chaotic response is obtained, then the calculations of both *n* and $T_{0}$ are not possible, as no homoclinic orbits occur, as illustrated in Figure 12.

Finally, in the identification interval 500≤t≤1000 used in this paper, we have 57, 205 and 254 points $C_{i}$ in Figure 10, Figure 11 and Figure 12, respectively. The number *n* was determined to be respectively 1, 5 and *undefined* for the three cases. Due to the long computation time required for the creation of two-parameter diagrams, all computations presented in this paper were done by using a 12-core computer and parallel algorithms described in the previous work [28].

## 5. Comparison with the Sample Entropy and 0–1 Test for Chaos Diagrams

**Figure 13:**
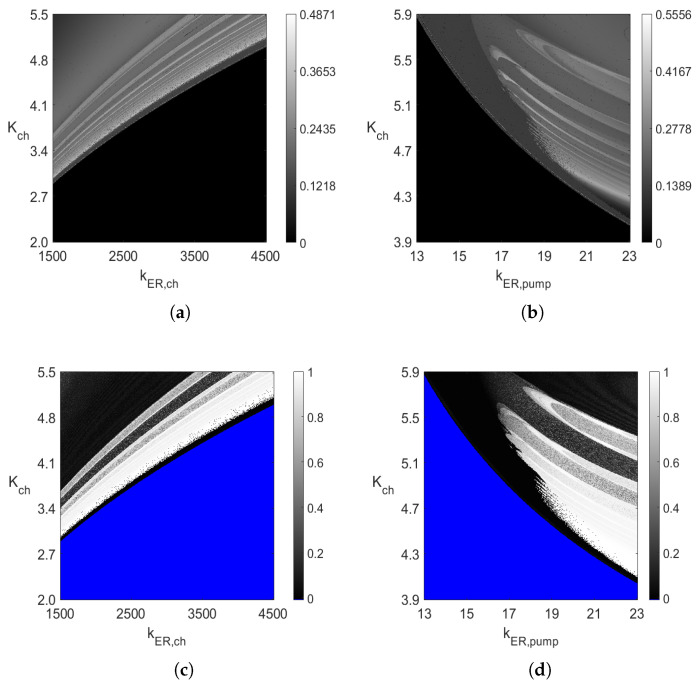
(**a**,**b**) Two-parameter 1000×1000 diagrams of the sample entropy values corresponding to the diagrams in Figure 3a,b, respectively; (**c**,**d**) two-parameter 1000×1000 diagrams of the 0–1 test for chaos (*K* values) corresponding to the diagrams in Figure 3a,b, respectively. The Runge–Kutta method of order 4 with constant step-size of integration 0.001 was used to solve (A3). The sample entropy diagrams were obtained with m=3, r=0.025 and N=10,000 (see [29]). The SE diagrams in (**a**,**b**) and the T01 diagrams in (**c**,**d**) were identified based on the solutions for 1000≤t≤2000 and 300≤t≤500, respectively. The parameter T=40 in the 0–1 test method (see [19,28]). (**a**) SE diagram with $k_{ER,pump}=20$. (**b**) SE diagram with $k_{ER,ch}=3500$. (**c**) T01 diagram with $k_{ER,pump}=20$.(**d**) T01 diagram with $k_{ER,ch}=3500$.

The diagrams in Figure 3a and Figure 4a are compared with the sample entropy (SE) [29,30] and 0–1 test (T01) [19,20] for chaos diagrams in Figure 13 in the same rectangular areas of the two varying parameters. The diagrams in Figure 13 are of size 1000 × 1000. Thus, system (A3) was solved $10^{6}$ times in the assumed rectangular areas. The sample entropy diagrams in Figure 13a,b show the values of sample entropy, between being very close to 0 for non-oscillatory value of parameters (black color) to the maximum values of 0.4871 and 0.5556 in the two figures. The higher the entropy value is, the more complex the oscillations are in Figure 3a and Figure 4a (period-n oscillations with large *n* value or chaotic oscillations). On the other hand, the two 0–1 test diagrams in Figure 13c,d have the *K* values close to 0 for periodic oscillations and the *K* values are close to 1 for chaotic ones. Notice the very good agreement between the nature of the diagrams in Figure 3a and Figure 4a and those in Figure 13.

## 6. Conclusions

Two types of two-parameter bifurcation diagrams (with the *period-n* responses and frequency distributions) were presented in this paper. Those diagrams provide useful information about the nature of the steady-state solutions of the nonlinear autonomous system of calcium oscillations with the mitochondria included. The same type of diagrams can be, in principle, created for any other nonlinear autonomous system with varying parameters. In general, because the two-parameter diagrams give different information about the changes in the steady-state responses than the one-parameter diagrams do, both kinds of diagrams complement each other. Our computations were done using parallel computing algorithms, since to create one diagram with two varying parameters requires solving the nonlinear autonomous system a few hundred thousands or even millions of times, depending on the two-parameter area and the resolution one desires. The identified possible chaotic oscillations occur in very narrow intervals of the parameter $k_{ER,ch}$ in Figure 1 and Figure 2 and scattered areas of white and green colors in Figure 3 and Figure 4, respectively. Those scatter areas of chaotic solutions are identical for both types of diagrams. Further comparisons with the sample entropy and 0–1 test for chaos diagrams also show a very good agreement of the obtained solutions.

The diagrams presented in this paper complement other types of one- and two-parameter diagrams one can create for nonlinear autonomous systems, such as, for example, those showing the Lyapunov exponents, Poincaré maps [31], the 0–1 test results [28] and entropy distribution [29,30].

## Data Availability

Not applicable.

## Appendix A. Model of Cytosolic Calcium Oscillations

One of the store models considered in the literature for complex $Ca^{2+}$ oscillations, with mitochondria included, is the model considered in [15,16,17,18]. The three compartments considered are: the cytosol, endoplasmic reticulum (ER) and mitochondria yielding the three dynamical variables, free Ca${}^{2+}$ concentration $Ca_{cyt}$, $Ca_{ER}$ and $Ca_{m}$, respectively (see Figure 1 in [16]). Denoting $x_{1}=Ca_{cyt}$, $x_{2}=Ca_{ER}$ and $x_{3}=Ca_{m}$, the nonlinear autonomous system is

(A1) $$\begin{array}{ccc} x_{1}^{\prime } & = & J_{ER,ch}-J_{ER,pump}+J_{ER,leak}+J_{CaPr}-J_{Pr}\\ \; & \; & +\frac{\rho_{m}}{\beta_{m}}\left(J_{m,out}-J_{m,in}\right)\\ x_{2}^{\prime } & = & \frac{\beta_{ER}}{\rho_{ER}}\left(J_{ER,pump}-J_{ER,leak}-J_{ER,ch}\right)\\ x_{3}^{\prime } & = & J_{m,in}-J_{m,out} \end{array}$$

where the ${}^{\prime }$ stands for the time derivative, and

(A2) $$\begin{array}{ccc} J_{ER,ch} & = & k_{ER,ch}\frac{x_{1}^{2}}{K_{ch}^{2}+x_{1}^{2}}\left(x_{2}-x_{1}\right)\\ J_{ER,pump} & = & k_{ER,pump}x_{1}\\ J_{ER,leak} & = & k_{ER,leak}\left(x_{2}-x_{1}\right)\\ J_{CaPr} & = & k_{-}CaPr\\ J_{Pr} & = & k_{+}x_{1}Pr\\ Ca_{tot} & = & x_{1}+\frac{\rho_{ER}}{\beta_{ER}}x_{2}+\frac{\rho_{m}}{\beta_{m}}x_{3}+CaPr\\ Pr_{tot} & = & Pr+CaPr\\ J_{m,in} & = & k_{m,in}\frac{x_{1}^{n}}{K_{m}^{n}+x_{1}^{n}}\\ J_{m,out} & = & k_{m,out}x_{3} \end{array}$$

with the parameters *n*, $k_{ER,ch}$, $K_{ch}$, $k_{ER,pump}$, $k_{ER,leak}$, $k_{-}$, $k_{+}$, $Ca_{tot}$, $\rho_{ER}$, $\beta_{ER}$, CaPr, $Pr_{tot}$, Pr, $\rho_{m}$, $\beta_{m}$, $k_{m,in}$, $k_{m,out}$ and $K_{m}$ being all positive. These parameters denote various physical quantities in the above model [16].

After substituting (A2) into (A1), we obtain

(A3) $$\begin{array}{ccc} x_{1}^{\prime } & = & k_{ER,ch}\frac{x_{1}^{2}}{K_{ch}^{2}+x_{1}^{2}}\left(x_{2}-x_{1}\right)-k_{ER,pump}x_{1}+\\ \; & \; & k_{ER,leak}\left(x_{2}\;-\;x_{1}\right)+\\ \; & \; & k_{-}\left(Ca_{tot}\;-x_{1}\frac{\rho_{ER}}{\beta_{ER}}x_{2}\;-\;\frac{\rho_{m}}{\beta_{m}}x_{3}\right)-\\ \; & \; & k_{+}\left(Pr_{tot}-Ca_{tot}+x_{1}+\frac{\rho_{ER}}{\beta_{ER}}x_{2}+\frac{\rho_{m}}{\beta_{m}}x_{3}\right)x_{1}+\\ \; & \; & \frac{\rho_{m}}{\beta_{m}}\left(k_{m,out}x_{3}-k_{m,in}\frac{x_{1}^{n}}{K_{m}^{n}+x_{1}^{n}}\right)\equiv {\tilde{f}}_{1}\left(x_{1},x_{2},x_{3},p\right)\\ x_{2}^{\prime } & = & \frac{\beta_{ER}}{\rho_{ER}}(k_{ER,pump}x_{1}-k_{ER,leak}\left(x_{2}-x_{1}\right)-\\ \; & \; & k_{ER,ch}\frac{x_{1}^{2}}{K_{ch}^{2}+x_{1}^{2}}\left(x_{2}-x_{1}\right))\equiv {\tilde{f}}_{2}\left(x_{1},x_{2},p\right)\\ x_{3}^{\prime } & = & k_{m,in}\frac{x_{1}^{n}}{K_{m}^{n}+x_{1}^{n}}-k_{m,out}x_{3}\equiv {\tilde{f}}_{3}\left(x_{1},x_{3},p\right) \end{array}$$

Notice the presence of many parameters denoted by the vector *p* in (A3) and the nonlinear terms of polynomial and rational types. All computations presented in this paper were done with the following parameters kept constant: n=8, $k_{ER,leak}$ = 0.05 s${}^{-1}$, $\rho_{ER}=0.01$, $\beta_{ER}=0.0025$, $k_{-}=0.01$ s${}^{-1}$, $k_{+}=0.1$ μM${}^{-1}$s${}^{-1}$, $Ca_{tot}=90$ μM, $Pr_{tot}=120$ μM, $\rho_{m}=0.01$, $\beta_{m}=0.0025$, $k_{m,in}=75$ μMs${}^{-1}$, $k_{m,out}=0.1265625$ s${}^{-1}$, $K_{m}=0.8$ μM. The varying parameters are identified in the bifurcation diagrams. The zero initial conditions for the three variables $x_{i}$, i=1,2,3, were used. For the one-parameter diagrams shown in Figure 1 and Figure 2, the *ode45* solver was used with the values dt=0.001 (the output step size), 0≤t≤500 (time horizon) and $abserr=relerr=10^{-8}$. The maximum values in the diagrams in Figure 1 and Figure 2 were identified with 750 discrete values of the horizontal parameter $k_{ER,ch}$ between its lower and upper bounds. For all two-parameter diagrams, the Runge–Kutta IV solver was used for 0≤t≤1000, with the number of maximum values and frequencies identified in the second half of that period.

## References

[1] Bendixson T. (1901). Sur les courbes définies par des équations defférentielles. Acta Math..

[2] Burton T.A. (2005). Volterra Integral and Differential Equations.

[3] Chouchane M., Amamou A. (2011). Bifurcation of limit cycles in fluid film bearings. Int. J. Non-Linear Mech..

[4] Varanis M.V., Tusset A.M., Balthazar J.M., Litak G., Oliveira C., Rocha R.T., Nabarrete A., Piccirillo V. (2020). Dynamics and control of periodic and non-periodic behavior of Duffing vibrating system with fractional damping and excited by a non-ideal motor. J. Franklin Inst..

[5] Aguilar-Lopez R., Martinez-Guerra R., Puebla H., Hernandez-Suarez R. (2010). High order sliding-mode dynamic control for chaotic intracellular calcium oscillations. Nonlinear Anal. Real World Appl..

[6] Marszalek W., Podhaisky H. (2016). 2D bifurcations and Newtonian properties of memristive Chua’s circuits. Europhys. Lett..

[7] Agarwal J., Blockley D.I., Woodman N.J. (1997). Qualitative analysis of non-linear dynamical systems. Comput. Methods Appl. Mech. Eng..

[8] Liao S. (2009). On the reliability of computed solutions of non-linear differential equations. Tellus Ser. A Dyn. Meteorol. Oceanogr..

[9] Nepomuceno E.G., Mendes E.M.A.M. (2017). On the analysis of pseudo-orbits of continuous chaotic nonlinear systems simulated using discretization schemes in a digital computer. Chaos Solitons Fractals.

[10] Nepomuceno E.G., Perc M. (2020). Computational chaos in complex networks. J. Complex Netw..

[11] Parker T.S., Chua L.O. (1989). Practical Numerical Algorithms for Chaotic Systems.

[12] Liu P., Liu X., Yu P. (2017). Mixed-mode oscillations in a three-store calcium dynamics model. Commun. Nonlinear Sci. Numer. Simulat..

[13] Hinrichsen D., Pritchard A.J. (2005). Mathematical Systems Theory I: Modelling, State Space Analysis. Stability and Robustness.

[14] Marszalek W. (2012). Circuits with oscillatory hierarchical Farey sequences and fractal properties. Circuits Syst. Signal Proc..

[15] Marhl M., Haberichter T., Brumen M., Heinrich R. (2000). Complex calcium oscillations and the role of mitochondria and cytosolic proteins. BioSystems.

[16] Grubelnick V., Larseb A.Z., Kummer U., Olsenb L.F., Marhl M. (2001). Mitochondria regulate the amplitude of simple and complex calcium oscillations. Biophys. Chem..

[17] Ji Q.-B., Lu Q.-S., Yang Z.-Q., Duan L.-X. (2008). Bursting Ca^2+^ oscillations and synchronization in coupled cells. Chin. Phys. Lett..

[18] Li X., Zhanga S., Liu X., Wang X., Zhou A., Liu P. (2018). Dynamic analysis on the calcium oscillation model considering the influences of mitochondria. BioSystems.

[19] Melosik M., Marszalek W. (2016). On the 0/1 test for chaos in continuous systems. Bull. Polish Acad. Sci. Tech. Sci..

[20] Marszalek W., Walczak M., Sadecki J. (2019). Testing deterministic chaos: Incorrect results of the 0-1 test and how to avoid them. IEEE Access.

[21] Goldbeter A. (1996). Biochemical Oscillations and Cellular Rhythms.

[22] Kovanis V., Gavrielides A., Gallas J.A.C. (2010). Labyrinth bifurcations in optically injected diode lasers. Eur. Phys. J. D.

[23] Rech P.C., Beims M.W., Gallas J.A.C. (2004). Naimark-Sacker bifurcations in linearly coupled quadratic maps. arXiv.

[24] Haapala A.F., Howell K.C. (2014). Representations of higher-dimensional Poincaré maps with applications to spacecraft trajectory design. Acta Astonautica.

[25] Ghikas D.P.K., Oikonomou F.D. (2018). Towards an information geometric characterization/classification of complex systems. I. Use of generalized entropies. Phys. A Stat. Mech. Its Appl..

[26] Gottwald G.A., Melbourne I. (2009). On the implementation of the 0-1 test for chaos. SIAM J. Appl. Dyn. Syst..

[27] Sadecki J., Marszalek W. (2019). Complex oscillations and two-parameter bifurcations of a memristive circuit with diode bridge rectifier. Microelectron. J..

[28] Walczak M., Marszalek W., Sadecki J. (2019). Using the 0-1 test for chaos in nonlinear continuous systems with two varying parameters: Parallel computations. IEEE Access.

[29] Richman J.S., Lake D.E., Moorman J.R. (2005). Sample entropy. Methods Enzymol..

[30] Chen B., Zhu Y., Hu J., Zhang M. (2010). On optimal estimations with minimum error entropy criterion. J. Franklin Inst..

[31] Pei L., Chen Y., Wang S. (2020). Complicated oscillations and non-resonant double Hopf bifurcation of multiple feedback delayed control system of the gut microbiota. Nonlinear Anal. Real World Appl..

